# Roadmap for naming uncultivated Archaea and Bacteria

**DOI:** 10.1038/s41564-020-0733-x

**Published:** 2020-06-08

**Authors:** Alison E. Murray, John Freudenstein, Simonetta Gribaldo, Roland Hatzenpichler, Philip Hugenholtz, Peter Kämpfer, Konstantinos T. Konstantinidis, Christopher E. Lane, R. Thane Papke, Donovan H. Parks, Ramon Rossello-Mora, Matthew B. Stott, Iain C. Sutcliffe, J. Cameron Thrash, Stephanus N. Venter, William B. Whitman, Silvia G. Acinas, Rudolf I. Amann, Karthik Anantharaman, Jean Armengaud, Brett J. Baker, Roman A. Barco, Helge B. Bode, Eric S. Boyd, Carrie L. Brady, Paul Carini, Patrick S. G. Chain, Daniel R. Colman, Kristen M. DeAngelis, Maria Asuncion de los Rios, Paulina Estrada-de los Santos, Christopher A. Dunlap, Jonathan A. Eisen, David Emerson, Thijs J. G. Ettema, Damien Eveillard, Peter R. Girguis, Ute Hentschel, James T. Hollibaugh, Laura A. Hug, William P. Inskeep, Elena P. Ivanova, Hans-Peter Klenk, Wen-Jun Li, Karen G. Lloyd, Frank E. Löffler, Thulani P. Makhalanyane, Duane P. Moser, Takuro Nunoura, Marike Palmer, Victor Parro, Carlos Pedrós-Alió, Alexander J. Probst, Theo H. M. Smits, Andrew D. Steen, Emma T. Steenkamp, Anja Spang, Frank J. Stewart, James M. Tiedje, Peter Vandamme, Michael Wagner, Feng-Ping Wang, Pablo Yarza, Brian P. Hedlund, Anna-Louise Reysenbach

**Affiliations:** 1 Division of Earth and Ecosystem Sciences, Desert Research Institute, Reno, NV USA; 2 Department of Evolution, Ecology and Organismal Biology, The Ohio State University, Columbus, OH USA; 3 Evolutionary Biology of the Microbial Cell, Department of Microbiology, Institut Pasteur, Paris, France; 4 Department of Chemistry and Biochemistry, Center for Biofilm Engineering, and Thermal Biology Institute, Montana State University, Bozeman, MT USA; 5 Australian Centre for Ecogenomics, School of Chemistry and Molecular Biosciences, The University of Queensland, Brisbane, Australia; 6 Department of Applied Microbiology, Justus-Liebig-Universität, Giessen, Germany; 7 School of Civil and Environmental Engineering, Georgia Tech, Atlanta, GA USA; 8 Department of Biological Sciences, University of Rhode Island, Kingston, RI USA; 9 Department of Molecular and Cellular Biology, University of Connecticut, Storrs, CT USA; 10 Mediterranean Institute for Advanced Studies, CSIC-UIB, Illes Balears, Spain; 11 School of Biological Sciences, University of Canterbury, Christchurch, New Zealand; 12 Department of Applied Sciences, Northumbria University, Newcastle upon Tyne, UK; 13 Department of Biological Sciences, University of Southern California, Los Angeles, CA USA; 14 Department of Biochemistry, Genetics and Microbiology, University of Pretoria, Pretoria, South Africa; 15 Department of Microbiology, University of Georgia, Athens, GA USA; 16 Department of Marine Biology and Oceanography, Institut de Ciènces del Mar, CSIC, Barcelona, Spain; 17 Max Planck Institute for Marine Microbiology, Bremen, Germany; 18 Department of Bacteriology, University of Wisconsin-Madison, Madison, WI USA; 19 CEA Technological Innovations for Detection and Diagnosis Laboratory, CEA Pharmacology and Immunoanalysis Unit (SPI), Bagnols-sur-Cèze, France; 20 Department of Marine Science, Marine Science Institute, University of Texas at Austin, Port Aransas, TX USA; 21 Department of Earth Sciences, University of Southern California, Los Angeles, CA USA; 22 Molecular Biotechnology, Department of Biosciences and Buchmann Institute for Molecular Life Sciences (BMLS), Goethe University Frankfurt, Frankfurt am Main, Germany; 23 Senckenberg Society for Nature Research, Frankfurt am Main, Germany; 24 Department of Microbiology and Immunology, Montana State University, Bozeman, MT USA; 25 University of the West of England, Bristol, England; 26 Department of Environmental Science, University of Arizona, Tuscon, AZ USA; 27 Bioscience Division, Los Alamos National Laboratory, Los Alamos, NM USA; 28 Microbiology Department, University of Massachusetts, Amherst, MA USA; 29 National Museum of Natural Sciences, CSIC, Madrid, Spain; 30 Escuela Nacional de Ciencias Biológicas, Instituto Politécnico Nacional, Mexico City, Mexico; 31 National Center for Agricultural Utilization Research, Crop Bioprotection Research Unit, Peoria, IL USA; 32 Department of Evolution and Ecology, Department of Medical Microbiology and Immunology, University of California, Davis, CA USA; 33 Bigelow Laboratory for Ocean Sciences, East Boothbay, ME USA; 34 Laboratory of Microbiology, Wageningen University & Research, Wageningen, the Netherlands; 35 LS2N, Université de Nantes, CNRS, Nantes, France; 36 Department of Organismic and Evolutionary Biology, Harvard University, Cambridge, MA USA; 37 GEOMAR-Helmholtz Centre for Ocean Research, RD3-Marine Ecology, RU-Marine Microbiology, Kiel, Germany; 38 Department of Marine Sciences, University of Georgia, Athens, GA USA; 39 Department of Biology, University of Waterloo, Waterloo, Canada; 40 Department of Land Resources and Environmental Sciences, Montana State University, Bozeman, MT USA; 41 School of Science, RMIT University, Melbourne, Victoria Australia; 42 School of Natural and Environmental Sciences, Newcastle University, Newcastle upon Tyne, UK; 43 School of Life Sciences, Sun Yat-Sen University, Guangzhou, China; 44 Department of Microbiology, University of Tennessee, Knoxville, TN USA; 45 Departments of Microbiology and Civil & Environmental Engineering, Center for Environmental Biotechnology, University of Tennessee, Knoxville, TN USA; 46 Biosciences Division, Oak Ridge National Laboratory, Oak Ridge, TN USA; 47 Centre for Microbial Ecology and Genomics, Department of Biochemistry, Genetics and Microbiology, University of Pretoria, Pretoria, South Africa; 48 Division of Hydrologic Sciences, Desert Research Institute, Las Vegas, NV USA; 49 Research Center for Bioscience and Nanoscience (CeBN), Japan Agency for Marine-Earth Science and Technology (JAMSTEC), Yokosuka, Japan; 50 School of Life Sciences, University of Nevada, Las Vegas, NV USA; 51 Astrobiology Center (CSIC-INTA), Madrid, Spain; 52 National Biotechnology Center, Cantoblanco, Madrid, Spain; 53 Department of Chemistry, Environmental Microbiology and Biotechnology, University of Duisburg-Essen, Essen, Germany; 54 Environmental Genomics and Systems Biology Research Group, Institute for Environment and Natural Resources, Zürich University for Applied Sciences (ZHAW), Wädenswil, Switzerland; 55 Departments of Microbiology and Earth and Planetary Sciences, University of Tennessee, Knoxville, TN USA; 56 Department of Biochemistry, Genetics and Microbiology, University of Pretoria, Pretoria, South Africa; 57 Department for Marine Microbiology and Biogeochemistry, Royal Netherlands Institute for Sea Research, Den Burg, the Netherlands; 58 Department of Cell and Molecular Biology, Uppsala University, Uppsala, Sweden; 59 Center for Microbial Ecology, Department of Plant, Soil and Microbial Sciences, Michigan State University, East Lansing, MI USA; 60 Department of Biochemistry and Microbiology, Ghent University, Gent, Belgium; 61 Centre for Microbiology and Environmental Systems Science, University of Vienna, Vienna, Austria; 62 International Center for Deep Life Investigation, School of Oceanography and School of Life Sciences and Biotechnology, Shanghai Jiao Tong University, Shanghai, China; 63 Ribocon GmbH, Bremen, Germany; 64 Biology Department, Portland State University, Portland, OR USA

**Keywords:** Archaea, Bacteria, Environmental microbiology

## Abstract

The assembly of single-amplified genomes (SAGs) and metagenome-assembled genomes (MAGs) has led to a surge in genome-based discoveries of members affiliated with Archaea and Bacteria, bringing with it a need to develop guidelines for nomenclature of uncultivated microorganisms. The International Code of Nomenclature of Prokaryotes (ICNP) only recognizes cultures as ‘type material’, thereby preventing the naming of uncultivated organisms. In this Consensus Statement, we propose two potential paths to solve this nomenclatural conundrum. One option is the adoption of previously proposed modifications to the ICNP to recognize DNA sequences as acceptable type material; the other option creates a nomenclatural code for uncultivated Archaea and Bacteria that could eventually be merged with the ICNP in the future. Regardless of the path taken, we believe that action is needed now within the scientific community to develop consistent rules for nomenclature of uncultivated taxa in order to provide clarity and stability, and to effectively communicate microbial diversity.

## Abstract (web summary)

In this Consensus Statement, the authors discuss the issue of naming uncultivated prokaryotic microorganisms, which currently do not have a formal nomenclature system due to a lack of type material or cultured representatives, and propose two recommendations including the recognition of DNA sequences as type material.

## Main

As new environments are explored and technological innovations improve tools for the characterization of microbial biodiversity, insights into bacterial and archaeal diversity are continually emerging^1,2^, including improved understanding of physiological capacity, ecology and evolution of organisms across the tree of life. These advances are based on both cultivation strategies^3,4^ and cultivation-independent methods that directly access diversity using single-cell^5,6^ or metagenomic sequencing^7–9^ (Box 1). Though our ability to culture fastidious microorganisms is improving, success seems to vary depending on the environment. For example, the microbial diversity of host-associated systems such as the human microbiome^11,12^ may be more amenable to cultivation compared to some environments such as soil. At present, it seems clear that most archaeal and bacterial diversity remains yet to be cultured^10,13^. The reasons are many, but as demonstrated recently by the cultivation of a member of the Asgard archaea^14^, syntrophic interactions, slow growth and media optimization can present formidable challenges.

Box 1 Glossary of terms

**Table:** 

**Term**	**Definition**
Single-amplified genome (SAG)	Assembled genome in which the DNA sequence is derived from a single microbial cell.
Metagenome-assembled genome (MAG)	Assembled genome from a heterogeneous assemblage of cells; the MAG may represent multiple populations (with minor sequence variants) and thus represents a species-level group rather than a single genetic variant—or strain—that is typically represented by a cultivated isolate.
International Code of Nomenclature of Prokaryotes (ICNP)	Typically referred to as ‘the Code’. The definitive set of rules, principles and recommendations for naming Bacteria and Archaea.
International Committee on Systematics of Prokaryotes (ICSP)	The body that oversees the nomenclature of prokaryotes and supervises the publication of the ICNP. Their Judicial Commission issues opinions concerning nomenclatural matters and revisions to the Code.
International Code of Nomenclature of Uncultivated Prokaryotes (ICNUP)	The Uncultivated Code that will be developed if plan B is adopted to circumscribe nomenclature for uncultivated organisms.
International Journal of Systematic and Evolutionary Microbiology (IJSEM)	The primary journal for publication of descriptions of novel microbial taxa and lists of valid names; also where proposals to rename taxa are submitted. The official publication of the ICSP and the Bacteriology and Applied Microbiology Division of the International Union of Microbiological Societies.
International Sequence Database Collaboration (INSDC)	International coalition of databases comprised of the National Center for Biotechnology Information, the European Bioinformatics Institute and the DNA Databank of Japan.
Genome Standards Consortium (GSC)	The GSC is an open-membership working body that enables genomic data integration, discovery and comparison through international community-driven standards.
Minimal information about a single-amplified genome (MISAG)	Standard developed by the GSC for reporting single-amplified genome assembly quality, completeness and contamination information along with additional metadata.
Minimal information about a metagenome-assembled genome (MIMAG)	Standard developed by the GSC for reporting metagenome-assembled genome assembly quality, completeness and contamination information along with additional metadata.
Systematics	Systematics is the study of the units of biodiversity and their relationships. This includes the discovery of the basic units of biodiversity (species), reconstructing the patterns of relationships of species at successively higher levels and building classifications based on these patterns and taxa. This term is often used synonymously with taxonomy.
Taxonomy	The branch of science concerned with the classification, identification and nomenclature pertaining to organisms, in particular.
Nomenclature	A system for giving names to organisms.
Classification	The placement of organisms into groups on the basis of evolutionary relationships as well as similarities and shared qualities or characteristics.
Etymology	In prokaryotic nomenclature, the etymology of a name is the semantic derivation of the Latinized name describing what it was based on.
Protologue	A protologue summarizes the new name, its etymology, the taxon properties and the designated type material for which the microorganism is known.

## Rules of prokaryotic nomenclature and current challenges

Describing biodiversity and identifying organisms are the scientific goals of taxonomy. Taxonomy integrates classification and nomenclature to describe biological diversity. Classification circumscribes and ranks taxa, and nomenclature is the process of assigning names. The commonly used Linnaean nomenclatural system focuses on the recognition of species as the basic unit, which are included in taxa of successively higher ranks (genus, family, order, class and phylum). There is some flexibility on how to circumscribe microbial species using phylogenetic, genotypic and phenotypic data. Once a species is delineated, rules of nomenclature given in the International Code of Nomenclature of Prokaryotes (ICNP or ‘the Code’^14^; see Box 2) guide the creation and assignment of names. This is true of all codes of nomenclature that currently exist—prokaryotes, viruses, animals, algae, fungi and plants—in addition to separate codes for cultivated plants and plant associations.

As the development of prokaryotic taxonomy has mainly been informed by cultivation, there is currently no mechanism in the Code to either assign rank, or formally name, members of Archaea or Bacteria discovered using cultivation-independent approaches. The absence of stable names for uncultivated prokaryotes results in confusion in the literature and stands in the way of an integrated classification system. Therefore, an urgent need exists to reconsider the rules of nomenclature to include the entirety of archaeal and bacterial diversity. By recognizing this challenge, the intent is not to dissuade efforts for cultivation; quite the contrary, there remains a crucial need in the field of microbiology to bring microorganisms into pure culture or stable co-culture, and for culture collections to provide material for all matters related to the study of living microorganisms (such as physiology, growth characteristics and cell division).

Box 2 Brief introduction to the ICNP “The primary purpose of giving a name to a taxon is to supply a means of referring to it rather than to indicate the characters or the history of the taxon.” is stated in principle 4 of the Code (ref.^15^). The Code requires that every name is associated to a type material, that all names are announced and that names that are validated first have priority. Names are validated either by direct publication of papers in IJSEM (summarized in notification lists) or, if published in another journal (referred to as ‘effective publication’), by submission of any appropriately documented request to IJSEM for inclusion of the name in the validation list. This process ensures that nomenclature rules are followed, that names are communicated to the scientific community and that redundancies are prevented for different taxa. The taxon description (or protologue) includes, at a minimum, the Latin etymology of the name, a brief description of the taxon, designation of type material and, currently, details of pure culture submission to two culture collections. Priority simply means that the first validly published legitimate name is the ‘correct’ name of a taxon.

Naming under the Code is hierarchical, that is, the names of the higher ranks are derived from the names of the lower ranks. For instance, the names for families and orders are based on a type genus, the names for classes are based upon a type order, the names for phyla are based upon a type class and, reversely, the type of a phylum is a class, the type of a class is an order, the type of an order is a genus and the type of a genus is a species. If a new MAG represents a new class then the genus and species would be named first, for example, ‘*Ca*. Caldatribacterium californiense’, in the family Caldatribacteriaceae, order Caldatribacteriales and class Caldatribacteria^4^. A consequence of this system is that even the highest ranks are based upon some type material, which is the evidence for the existence of the taxon. Thus, this hierarchical system communicates phylogenetic information and differing levels of relatedness. If a future integrated Code for cultured and uncultivated organisms can be adopted, synonymies will be avoided. The requirement to deposit viable pure cultures into two international culture collections as type material is a relatively recent development in taxonomy. Prior to changes to the Code initiated in 1996, detailed descriptions or nonviable specimens were admissible as type material, as is currently the standard in other codes of nomenclature (such as the International Code of Nomenclature for algae, fungi, and plants; International Code of Zoological Nomenclature; and The International Code of Virus Classification and Nomenclature).

## Naming conventions

Thus far, two conventions have been used to name uncultivated taxa. The first applies alphanumeric identifiers to 16 S ribosomal RNA gene sequence clusters (for example, SAR11), which is now being extended to metagenome-assembled genomes (MAGs) and single-amplified genomes (SAGs). Alphanumeric identifiers are convenient as placeholders and can be used for communicating the underlying taxonomic or phylogenetic relationships and organizing diversity. However, the lack of consensus amongst the scientific community on rules for alphanumerical identifiers has resulted in frequent synonymies and confusion in the literature^15^. Secondly, an International Committee on Systematics of Prokaryotes (ICSP)-sanctioned approach for naming uncultivated taxa under the provisional ‘*Candidatus*’ classification has been in place for more than two decades^15^. However, *Candidatus* is a category with no standing in nomenclature; thus, *Candidatus* names do not necessarily complement official nomenclature and do not have priority—that is, they do not have to be retained if representatives of the taxon are subsequently cultivated.

This Consensus Statement proposes two potential paths to develop a system of nomenclature for uncultivated microorganisms that allows them to be classified and named with a high degree of fidelity using MAG and/or SAG sequences. This would allow these microorganisms to be described according to predicted characteristics, and to be linked to environmental and ecological contextual data, resulting in similar integrity and reproducibility to the current system used to name and classify cultivated microorganisms^17^. One path requires modifications to the Code to allow the use of DNA sequence data as ‘type material’ as proposed by Whitman^18^, whereas the other creates a parallel code for uncultivated microorganisms, as previously proposed^16,19^. The concept of type material in these cases reflects that DNA sequence deposited in an International Sequence Database Collaboration repository is the informational entity, supplanting the current ICNP requirement to deposit viable cultures in at least two culture collections. The focus of this Consensus Statement pertains to cases in which Archaea or Bacteria that are represented by DNA sequence information are to be named formally (at all levels of the taxonomic rank appropriate for the microorganism to be described), and where descriptive protologues are generated based on the DNA sequence information and supporting cultivation-independent and environmental data^20^. This Consensus Statement does not specifically address the overwhelming abundance of MAG and SAG data that will not be formally named, that is, those that are not studied in sufficient detail to provide meaningful insight into their structural, physiological, ecological or evolutionary properties. However, we advocate the adoption of quality standard frameworks^21^ for both formally named and alphanumerically identified MAGs and SAGs.

## Lessons from the history of prokaryotic taxonomy

Prokaryotic taxonomy underwent two revolutions in the latter half of the twentieth century that, we posit, are analogous to the present situation. Initially, methodological limitations in archaeal and bacterial classification, particularly the reliance on staining, morphology and physiological properties, led to a confusing proliferation of names and a poorly ordered taxonomy rife with synonyms^22^. Consequently, an ICSP ad hoc committee was appointed in 1973 to review the legitimate names of bacteria and compile the Approved Lists of Bacterial Names, which designated type strains, and, in some cases, type material (the description, illustration or preserved specimen), with valid names^23^. Names not included in the list lost their standing in nomenclature (Box 1).

Several years later, in 1987 and 1990, two ad hoc committees discussed the incorporation of DNA sequence information into the bacterial species definition which resulted in the integrated use of phylogenetic and phenotypic characteristics, or polyphasic taxonomy^24^. In 2002, a fourth ad hoc committee revisited the species definition in light of new molecular-sequence-based methods, encouraged the use of the *Candidatus* provisional category and recommended data standards utilizing sequence databases^25^. Since then, no additional committees have been appointed to address the opportunities and complexities of massive increases in genomic data provided by advancements in DNA sequencing technology.

## Stabilizing *Candidatus* names

At present, there is a need to formally account for all *Candidatus* taxa that have been described according to the original proposal^15^; such an effort is already underway^26^. Since 1995, more than 700 *Candidatus* names have been proposed but, due to the lack of official rules and oversight, a significant proportion do not comply with the Code^27^. Many names have not been captured in a unified list; some names lack key aspects of the description such as the designation of a type or an etymology, and the quality of available data to serve as type material for *Candidatus* taxa varies greatly. For instance, some *Candidatus* species are only linked to 16 S rRNA gene sequences, or to no genetic data at all, which complicates linking legacy with modern datasets. In addition, there are numerous higher taxa (such as candidate phyla) for which no lower rank or type has been designated. This also contradicts the principles of all codes of taxonomic nomenclature. Naming higher taxa has become common practice (compounded by the problem that the rank of phylum currently lacks official status in the Code^28^), particularly for newly discovered uncultivated lineages^17^.

## The path forward

**Fig. 1:**
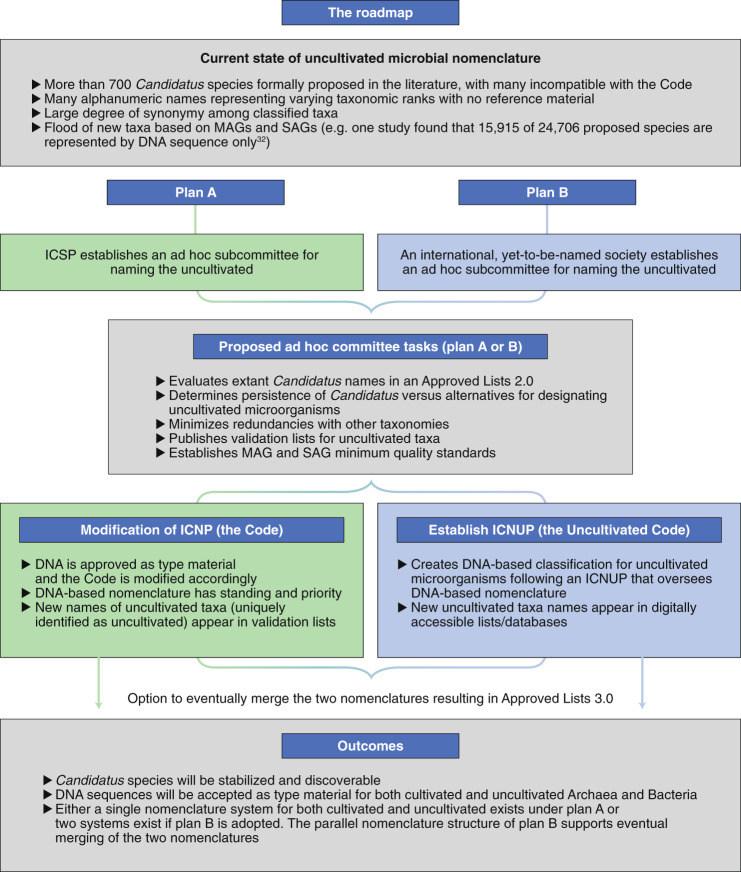
Proposed roadmaps for nomenclature of uncultivated Archaea and Bacteria. Plans A and B provide two alternatives for inclusion of uncultivated Archaea and Bacteria into the classical Linnaean nomenclature system.

The path described is the result of engagement with a large consortium of scientists who provided input (both co-authors and endorsees; see Supplementary Table 1). Plan A proposes the formal revision of the Code to include uncultivated organisms represented by DNA sequence information as the nomenclatural type^18^, albeit with an allowance to distinguish cultivated and uncultivated taxa^17^ (Fig. 1). Here, we refer to microorganisms available in pure culture (or stable co-culture) that can be named according to the rules of the Code as ‘cultivated’, and those that are recognized through their DNA sequence information as ‘uncultivated’ (including mixed cultures in which the members are recognized through DNA sequence information). Plan A could be initiated by establishing a subcommittee of the ICSP to review and stabilize the current *Candidatus* nomenclature, develop standards for DNA sequence data to serve as type material, address the use of *Candidatus* or other alternatives (such as superscripts ^u^, ^c^ or ^e^ to represent uncultivated, *Candidatus* or environmental, respectively) to distinguish between names of uncultivated organisms versus those derived from cultures^15,17^ and to establish an updated list (an ‘Approved Lists 2.0’) of approved nomenclature to include previously named taxa with DNA sequence as type material. These new names and descriptions (protologues including etymology, taxon properties, inferred phenotype and sequence deposit accession numbers), whether for single taxa or large-scale MAG and SAG studies, would be communicated through the literature, reviewed by the International Journal of Systematic and Evolutionary Microbiology (IJSEM) list editors and then included in the revised list, granting them priority over subsequent names. Plan A creates a framework within the Code that will lead to a smooth integration of a harmonized nomenclature and will facilitate future unified nomenclature.

An alternate, near-term solution (plan B) would be the creation of a parallel code for uncultivated taxa (the ‘Uncultivated Code’; Fig. 1) under the auspices of an international entity with enough authority to provide a unified framework. This entity could take on the responsibility for supporting the development of an International Code of Nomenclature of Uncultivated Prokaryotes (ICNUP; that is, the Uncultivated Code) ruling on its actions and publishing a list (a digital record) of valid names for uncultivated taxa. The ICNUP would appoint an ad hoc committee to address the current *Candidatus* names and develop an ‘Approved Uncultivated Lists 2.0’ inclusive of *Candidatus* species names to stabilize the nomenclature and ascertain priority. The *Candidatus* designation could be preserved, or some other notation recommended to identify uncultivated status. Likewise, the ad hoc committee could provide guidelines regarding quality standards and full taxonomic classification for MAGs and SAGs to be named going forward, possibly with input from the Genome Standards Consortium (GSC). As recommended^15^, the rules of the Uncultivated Code would be analogous to the Code, and *Candidatus* names already published and supported by DNA sequence information would be granted priority as in plan A. This parallel structure allows the two nomenclature systems to be merged to yield a single, unified collection of validly published names (for example, an ‘Approved Lists 3.0’) if and when supported by the scientific community. Alternatively, the two systems could exist in parallel and never be unified (Fig. 1). We also recommend that names established under the Uncultivated Code be conserved in cases where uncultivated taxa are brought into pure culture—the ultimate path for microbiological characterization.

**Fig. 2:**
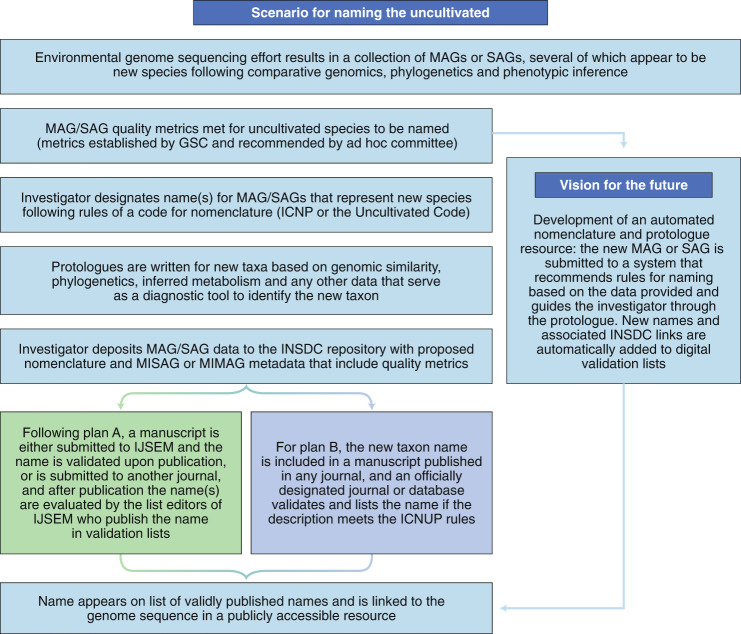
Scenario for naming uncultivated Archaea and Bacteria. In cases where naming a new species is warranted, the steps outlined here are a likely process for nomenclature regardless of whether plan A or plan B (Fig. 1) is adopted.

Plan A works within the Code to avoid decentralizing the process of nomenclature—thus mitigating disputes over priority in the future—and could be implemented rapidly to effectively meet the immediate demands of the scientific community. However, a practical, expedient solution is required. If ratification of the revised Code via the ICSP is prolonged (as it has been recently^16^), adoption of the scenario described in plan B could provide a timely solution to avoid conflict in the nomenclatural system and promote communication across stakeholders in the prokaryotic sciences. In a practical sense, both plans result in a similar process for naming uncultivated microorganisms in which the uncultivated representatives have a unique identifier (Fig. 2).

## Quality standards and digital protologues

Regardless of the path forward (plan A or B), we propose the development of genomic standards to guide the naming of uncultivated taxa to the extent possible, across all taxonomic ranks. Relevant standards for MAGs and SAGs have recently been published, including recommendations on contextual information or metadata (for example, geographic location, biome and sampled material characteristics)^16^, minimal standards based on MAG and SAG completeness and contamination^21^, and type material^17^ (Table 1). In addition, the overseeing body (such as the ICSP) could also provide direction to the scientific community on how and when to name (and not name) a MAG or SAG. Likewise, the overseeing body could also recommend standardized naming practices that could be applied to high quality MAGs and SAGs currently deposited in public repositories.

**Table 1:** Data inputs for an ad hoc committee naming uncultivated SAGs and MAGs

**Category**	**Parameters for data quality recommendations**
Genome quality	Percent completion
	Percent contamination
	Presence of 5S, 16S 23S rRNA gene (and level of completeness)
	Number of tRNA genes
Assembly quality	N50 (defined as the length of the shortest contig in the set of largest contigs that together constitute at least half of the total assembly size)
	Number of contigs
Naming conventions	Status of *Candidatus* and future nomenclature (for example, superscript ^u^, ^c^ or ^e^ designating uncultivated, *Candidatus* or environmental microorganisms, respectively)
Description requirements	Phenotype or metabolic prediction based on DNA sequence
	Ecological and biogeographic consideration
	Additional metadata (including guanine and cytosine content, genome size and number of protein coding genes)
Validation of MAG and SAG nomenclature	Potential for informational system of classification, validation and DOI assignment

With the impending adoption of minimal information about a single-amplified genome (MISAG) and metagenome-assembled genome (MIMAG) checklists in GenBank and the European Nucleotide Archive^29^, it is now up to the scientific community, through peer review and journal policies, to ensure reporting of SAG and MAG data quality. While MAGs may not always represent single genomes, if they are of high quality and have minimal contamination, they likely represent the consensus genome of a natural microbial population. Thus, while the designation of a type strain is unlikely (albeit advances in long-read sequencing technology may aid in this respect), MAGs can act as the nomenclatural type for a species despite their mosaic nature. This distinction should be carried forward regardless of whether plan A or B are adopted. If standards are not enforced by the scientific community, the risk is that poor-quality genomes with contaminating sequences could exacerbate transitive errors in annotation (such as cases in which a contaminating sequence could be misidentified as being associated with the particular MAG) and species assignments in downstream phylogenomic studies—a clearly undesirable situation that is not limited to MAGs and SAGs.

Current publishing capabilities will continue to struggle to keep pace with the anticipated number of taxonomic descriptions, especially if MAGs and SAGs were allowed as type material. Therefore, the future of this field requires breakthroughs in information access and advances in database interoperability. Examples of these breakthroughs include the creation of standardized, machine-readable formats for nomenclature that can capture name changes, automated taxonomic assignment based on big-data analysis (with best criteria discussed and widely adopted in the community) and nomenclature pipelines guiding the user through rules for naming by following guidelines of the Code or the Uncultivated Code. Automated mechanisms to create properly formatted protologues (Fig. 2) are also urgently needed^9,30,31^.

## Concluding remarks

This Consensus Statement addresses the need to provide a stable nomenclature and taxonomy for uncultivated Archaea and Bacteria that will enable scientific discourse among the many fields that communicate microbial diversity information. The proposed plans (A or B) enable a roadmap for communicating the enormous diversity of the prokaryotic world. This includes a standardized framework for naming uncultivated Archaea and Bacteria that will provide a needed structure to the classification system and allow for scientific communication regarding diversity across the microbial sciences. The proposed roadmap is not meant to suggest that all MAGs and SAGs will be named according to the Linnaean nomenclature—many will remain with alphanumeric identifiers. Instead, the roadmap provides a path for naming MAGs and SAGs that meet high quality standards. There are additional needs for discovering and classifying both named and unnamed MAGs and SAGs based on phylogenomics, and for identifying high-quality, well-curated representative (or ‘type’) genomes^32^ that are not addressed here.

Regardless, implementation of either of our proposed plans will require engagement from the scientific community (including the ICSP) to address the finer details, some of which were not captured herein. As evidenced by our effort here, there is substantial interest from the scientific community to participate in the decision-making process for determining standards in nomenclature that affect the entire microbiology field. We can look to the virus community for guidance in their adoption of nomenclature rules based on viral genome sequences^33^ in which the International Committee on Taxonomy of Viruses endorsed the proposal to include (meta)genome sequence data. The utility of DNA sequences as type material is relevant to organisms across the tree of life and biologists in other fields, including fungi and protists, face similar challenges^34^. We hope that the solutions identified in this roadmap might also apply to the naming of other organisms in these diverse fields.

*Note added in proof:* Whilst this manuscript was in revision, the ICSP held an e-mail discussion forum, followed by voting on the Whitman^18^ proposals to modify the ICNP to allow sequence data as type material (plan A). In the subsequent ICSP vote, these proposals were rejected. Minutes of the e-mail discussion will be made available on the ICSP website. Although further proposals to modify the ICNP may be forthcoming, this result makes the imminent adoption of plan A unlikely and therefore increases the likelihood of plan B being enacted.

## Supplementary information

Supplementary Information Supplementary Table 1.
