# Author Correction: Roadmap for naming uncultivated Archaea and Bacteria

**DOI:** 10.1038/s41564-020-00827-2

**Published:** 2020-11-12

**Authors:** Alison E. Murray, John Freudenstein, Simonetta Gribaldo, Roland Hatzenpichler, Philip Hugenholtz, Peter Kämpfer, Konstantinos T. Konstantinidis, Christopher E. Lane, R. Thane Papke, Donovan H. Parks, Ramon Rossello-Mora, Matthew B. Stott, Iain C. Sutcliffe, J. Cameron Thrash, Stephanus N. Venter, William B. Whitman, Silvia G. Acinas, Rudolf I. Amann, Karthik Anantharaman, Jean Armengaud, Brett J. Baker, Roman A. Barco, Helge B. Bode, Eric S. Boyd, Carrie L. Brady, Paul Carini, Patrick S. G. Chain, Daniel R. Colman, Kristen M. DeAngelis, Maria Asuncion de los Rios, Paulina Estrada-de los Santos, Christopher A. Dunlap, Jonathan A. Eisen, David Emerson, Thijs J. G. Ettema, Damien Eveillard, Peter R. Girguis, Ute Hentschel, James T. Hollibaugh, Laura A. Hug, William P. Inskeep, Elena P. Ivanova, Hans-Peter Klenk, Wen-Jun Li, Karen G. Lloyd, Frank E. Löffler, Thulani P. Makhalanyane, Duane P. Moser, Takuro Nunoura, Marike Palmer, Victor Parro, Carlos Pedrós-Alió, Alexander J. Probst, Theo H. M. Smits, Andrew D. Steen, Emma T. Steenkamp, Anja Spang, Frank J. Stewart, James M. Tiedje, Peter Vandamme, Michael Wagner, Feng-Ping Wang, Pablo Yarza, Brian P. Hedlund, Anna-Louise Reysenbach

**Affiliations:** 1 Division of Earth and Ecosystem Sciences, Desert Research Institute, Reno, NV USA; 2 Department of Evolution, Ecology and Organismal Biology, The Ohio State University, Columbus, OH USA; 3 Evolutionary Biology of the Microbial Cell, Department of Microbiology, Institut Pasteur, Paris, France; 4 Department of Chemistry and Biochemistry, Center for Biofilm Engineering, and Thermal Biology Institute, Montana State University, Bozeman, MT USA; 5 Australian Centre for Ecogenomics, School of Chemistry and Molecular Biosciences, The University of Queensland, Brisbane, Australia; 6 Department of Applied Microbiology, Justus-Liebig-Universität, Giessen, Germany; 7 School of Civil and Environmental Engineering, Georgia Tech, Atlanta, GA USA; 8 Department of Biological Sciences, University of Rhode Island, Kingston, RI USA; 9 Department of Molecular and Cellular Biology, University of Connecticut, Storrs, CT USA; 10 Mediterranean Institute for Advanced Studies, CSIC-UIB, Illes Balears, Spain; 11 School of Biological Sciences, University of Canterbury, Christchurch, New Zealand; 12 Department of Applied Sciences, Northumbria University, Newcastle upon Tyne, UK; 13 Department of Biological Sciences, University of Southern California, Los Angeles, CA USA; 14 Department of Biochemistry, Genetics and Microbiology, University of Pretoria, Pretoria, South Africa; 15 Department of Microbiology, University of Georgia, Athens, GA USA; 16 Department of Marine Biology and Oceanography, Institut de Ciènces del Mar, CSIC, Barcelona, Spain; 17 Max Planck Institute for Marine Microbiology, Bremen, Germany; 18 Department of Bacteriology, University of Wisconsin-Madison, Madison, WI USA; 19 CEA Technological Innovations for Detection and Diagnosis Laboratory, CEA Pharmacology and Immunoanalysis Unit (SPI), Bagnols-sur-Cèze, France; 20 Department of Marine Science, Marine Science Institute, University of Texas at Austin, Port Aransas, TX USA; 21 Department of Earth Sciences, University of Southern California, Los Angeles, CA USA; 22 Molecular Biotechnology, Department of Biosciences and Buchmann Institute for Molecular Life Sciences (BMLS), Goethe University Frankfurt, Frankfurt am Main, Germany; 23 Senckenberg Society for Nature Research, Frankfurt am Main, Germany; 24 Department of Microbiology and Immunology, Montana State University, Bozeman, MT USA; 25 University of the West of England, Bristol, England; 26 Department of Environmental Science, University of Arizona, Tuscon, AZ USA; 27 Bioscience Division, Los Alamos National Laboratory, Los Alamos, NM USA; 28 Microbiology Department, University of Massachusetts, Amherst, MA USA; 29 National Museum of Natural Sciences, CSIC, Madrid, Spain; 30 Escuela Nacional de Ciencias Biológicas, Instituto Politécnico Nacional, Mexico City, Mexico; 31 National Center for Agricultural Utilization Research, Crop Bioprotection Research Unit, Peoria, IL USA; 32 Department of Evolution and Ecology, Department of Medical Microbiology and Immunology, University of California, Davis, CA USA; 33 Bigelow Laboratory for Ocean Sciences, East Boothbay, ME USA; 34 Laboratory of Microbiology, Wageningen University & Research, Wageningen, the Netherlands; 35 LS2N, Université de Nantes, CNRS, Nantes, France; 36 Department of Organismic and Evolutionary Biology, Harvard University, Cambridge, MA USA; 37 GEOMAR-Helmholtz Centre for Ocean Research, RD3-Marine Ecology, RU-Marine Microbiology, Kiel, Germany; 38 Department of Marine Sciences, University of Georgia, Athens, GA USA; 39 Department of Biology, University of Waterloo, Waterloo, Canada; 40 Department of Land Resources and Environmental Sciences, Montana State University, Bozeman, MT USA; 41 School of Science, RMIT University, Melbourne, Victoria Australia; 42 School of Natural and Environmental Sciences, Newcastle University, Newcastle upon Tyne, UK; 43 School of Life Sciences, Sun Yat-Sen University, Guangzhou, China; 44 Department of Microbiology, University of Tennessee, Knoxville, TN USA; 45 Departments of Microbiology and Civil & Environmental Engineering, Center for Environmental Biotechnology, University of Tennessee, Knoxville, TN USA; 46 Biosciences Division, Oak Ridge National Laboratory, Oak Ridge, TN USA; 47 Centre for Microbial Ecology and Genomics, Department of Biochemistry, Genetics and Microbiology, University of Pretoria, Pretoria, South Africa; 48 Division of Hydrologic Sciences, Desert Research Institute, Las Vegas, NV USA; 49 Research Center for Bioscience and Nanoscience (CeBN), Japan Agency for Marine-Earth Science and Technology (JAMSTEC), Yokosuka, Japan; 50 School of Life Sciences, University of Nevada, Las Vegas, NV USA; 51 Astrobiology Center (CSIC-INTA), Madrid, Spain; 52 National Biotechnology Center, Cantoblanco, Madrid, Spain; 53 Department of Chemistry, Environmental Microbiology and Biotechnology, University of Duisburg-Essen, Essen, Germany; 54 Environmental Genomics and Systems Biology Research Group, Institute for Environment and Natural Resources, Zürich University for Applied Sciences (ZHAW), Wädenswil, Switzerland; 55 Departments of Microbiology and Earth and Planetary Sciences, University of Tennessee, Knoxville, TN USA; 56 Department of Biochemistry, Genetics and Microbiology, University of Pretoria, Pretoria, South Africa; 57 Department for Marine Microbiology and Biogeochemistry, Royal Netherlands Institute for Sea Research, Den Burg, the Netherlands; 58 Department of Cell and Molecular Biology, Uppsala University, Uppsala, Sweden; 59 Center for Microbial Ecology, Department of Plant, Soil and Microbial Sciences, Michigan State University, East Lansing, MI USA; 60 Department of Biochemistry and Microbiology, Ghent University, Gent, Belgium; 61 Centre for Microbiology and Environmental Systems Science, University of Vienna, Vienna, Austria; 62 International Center for Deep Life Investigation, School of Oceanography and School of Life Sciences and Biotechnology, Shanghai Jiao Tong University, Shanghai, China; 63 Ribocon GmbH, Bremen, Germany; 64 Biology Department, Portland State University, Portland, OR USA

**Keywords:** Archaea, Bacteria, Environmental microbiology

Correction to: *Nature Microbiology* 10.1038/s41564-020-0733-x, published online 8 June 2020.

In the version of this Consensus Statement originally published, Pablo Yarza was mistakenly not included in the author list. Also, in Supplementary Table 1, Alexander Jaffe was missing from the list of endorsees. These errors have now been corrected and the updated Supplementary Table 1 is available online.

## Supplementary information

Supplementary Information

