# Supplementary material for: Roadmap for naming uncultivated Archaea and Bacteria
Source: Nat Microbiol. 2020 Jun 8;5(8):987–94. doi: 10.1038/s41564-020-0733-x (PMC7381421; doi:10.1038/s41564-020-0733-x)
Supplement: Supplementary file 1 — Supplementary Table 1. [file 41564_2020_733_MOESM1_ESM.pdf]

https://doi.org/10.1038/s41564-020-0733-x

Supplementary information

Roadmap for naming uncultivated Archaea and Bacteria

In the format provided by the authors and unedited

Supplementary Information

Table S1. List of endorsees of the Roadmap plans A or B.

Endorsee Name	Institution	Country
Amils, Ricardo	CBMSO (CSIC, UAM), Autonomous University of Madrid, Madrid	Spain
Anton, Josefa	Department of Physiology, Genetics and Microbiology, University of Alicante, San Vicente del Raspeig	Spain
Beukes, Chrizelle W	Department of Biochemistry, Genetics and Microbiology, Forestry and Agricultural Biotechnology Institute (FABI), University of Pretoria	South Africa
Brochier, Celine	Laboratory of Biometrics and Biological Evolution, University of Lyon, Lyon	France
Casamayor, Emilio	Spanish Research Council (CSIC), Center for Advanced Studies of Blanes (CEAB), Blanes	Spain
Cole, James	Department of Plant, Soil and Microbial Sciences, Michigan State University, East Lansing, MI	USA
Colwell, Frederick	College of Earth, Ocean, and Atmospheric Sciences, Oregon State University, Corvalis, OR	USA
de la Torre, José R	Department of Biology, San Francisco State University, San Francisco, CA	USA
de Lorenzo, Víctor	National Centre for Biotechnology, Madrid	Spain
de Maayer, Pieter	School of Molecular and Cell Biology, University of the Witwatersrand, Johannesburg	South Africa
Dedysh, Svetlana	Winogradsky Institute of Microbiology, Research Center of Biotechnology, Russian Academy of Sciences, Moscow, Russia	Russia
DeLong, Edward F	Department of Oceanography, University of Hawaii Manoa, Honolulu, HI	USA
Dodsworth, Jeremy A	Department of Biology, California State University, San Bernardino, CA	USA
Dubilier, Nicole	Max Planck Institute for Marine Microbiology, Bremen	Germany
Eloe-Fadrosh, Emiley	DOE Joint Genome Institute, Lawrence Berkeley National Laboratory, Berkeley, CA	USA
Eme, Laura	CNRS & University of Paris-Sud, Paris	France

Endorsee Name	Institution	Country
Fierer, Noah	Department of Ecology & Evolutionary Biology, University of Colorado, Boulder, CO	USA
Fournier, Pierre-Edouard	UMR VITROME, Méditerranée Infection, Univ Marseille, Marseille	France
Glöckner, Frank Oliver	Computing and Data Centre, Alfred Wegener Institute - Helmholtz Center for Polar- and Marine Research	Germany
Giovanonni, Stephen	Department of Microbiology, Oregon State University, Corvallis OR	USA
Gonzalez-Pastor, José Eduardo	Department of Molecular Evolution, Centro de Astrobiología (CSIC-INTA), Madrid	Spain
Gonzalez, Bernardo	Faculty of Engineering and Science, University Adolfo Ibañez, Santiago	Chile
Goodfellow, Mike	School of Natural and Environmental Sciences, Newcastle University, Newcastle upon Tyne	UK
Gupta, Radhey	Department of Biochemistry and Biomedical Sciences, McMaster University, Hamilton, ON	Canada
Hahn, Martin	Department for Limnology, University Innsbruck, Innsbruck	Austria
Hallam, Steven	Department of Microbiology and Immunology, University of British Columbia, Vancouver, BC	Canada
Herbold, Craig W	University of Vienna, Centre for Microbiology and Environmental Systems Science, Vienna	Austria
Jaffe, Alexander	University of California Berkeley, Dep. of Earth and Planetary Science, Berkeley, CA	USA
Joye, Samantha B	Department of Marine Sciences, University of Georgia, Athens, GA	USA
Kirby, Bronwyn M	Institute for Microbial Biotechnology and Metagenomics, University of the Western Cape, Cape Town	South Africa
Koonin, Eugene	National Center for Biotechnology Information (NCBI), National Library of Medicine (NLM), National Institutes of Health (NIH) Bethesda, MD	USA
Kormas, Konstantinos	Department of Ichthyology & Aquatic Environment, University of Thessaly	Greece
Kyrpides, Nikos	DOE Joint Genome Institute, Lawrence Berkeley National Laboratory, Berkeley, CA	USA

Endorsee Name	Institution	Country
L'Haridon, Stephane	University of Brest, European Institute for Marine Studies, Laboratory of the Microbiology of the Extreme Environments, Plouzané	France
LaRowe, Douglas E	Department of Earth Sciences, University of Southern California, Los Angeles, CA	USA
López-García, Purificación	CNRS & University of Paris-Saclay, Orsay	France
Ludwig, Wolfgang	Max Planck Institute for Marine Microbiology, Bremen	Germany
Nealson, Kenneth H	Department of Earth Sciences, University of Southern California, Los Angeles, CA	USA
Orcutt, Beth N	Bigelow Laboratory for Ocean Sciences, East Boothbay, ME	USA
Orphan, Victoria J	Division of Geological and Planetary Sciences, California Institute of Technology, Pasadena, CA	USA
Ramos, Juan L	Zaidín Experimental Station, CSIC, Granada, Spain	Spain
Sangal, Vartul	Faculty of Health and Life Sciences, Northumbria University, Newcastle upon Tyne	UK
Santoro, Alyson E	Department of Ecology, Evolution and Marine Biology, University of California, Santa Barbara	USA
Schink, Bernhard	Department of Biology, University Konstanz, Konstanz	Germany
Schleifer, Karl H	Department of Microbiology, Technical University, Munich	Germany
Schleper, Christa	Archaea Biology and Ecogenomics Division, University of Vienna, Vienna	Austria
Schoch, Conrad L	National Center for Biotechnology Information (NCBI), National Library of Medicine (NLM), National Institutes of Health (NIH) Bethesda, MD	USA
Shouche, Yogest	National Centre for Microbial Resource, National Centre for Cell Science, Pune	India
Swings, Jean	Laboratory for Microbiology, University of Gent	Belgium
Takai, Ken	Institute for Extra-cutting-edge Science and Technology Avant-garde Research (X-star), Japan Agency for Marine-Earth Science & Technology (JAMSTEC), Yokosuka	Japan
Teske, Andreas	Department of Marine Sciences, University of North Carolina, Chapel Hill, NC	USA

Endorsee Name	Institution	Country
Thompson, Fabiano	SAGE/COPPE, Federal University of Rio de Janeiro, Rio de Janeiro	Brazil
Thormann, Kai M	Department for Microbiology and Molecular Biology, University Giessen, Justus-Liebig-Universität Gießen, Gießen	Germany
Wade, William	Centre for Host-Microbiome Interactions, King's College London, London	UK
Wilharm, Gottfried	Robert Koch Institute, Wernigerode	Germany
Woyke, Tanja	DOE Joint Genome Institute, Lawrence Berkeley National Laboratory, Berkeley, CA	USA
